# Shape Memory Characteristics of Injection Molded Poly(lactic acid) Multiscale Hybrid Composites

**DOI:** 10.1021/acsomega.4c06592

**Published:** 2024-11-15

**Authors:** Balázs Tatár, László Mészáros

**Affiliations:** † Department of Polymer Engineering, Faculty of Mechanical Engineering, Budapest University of Technology and Economics, Műegyetem rkp. 3, H-1111 Budapest, Hungary; ‡ HUN-REN Research Group for Composite Science and Technology, Műegyetem rkp. 3, H-1111 Budapest, Hungary

## Abstract

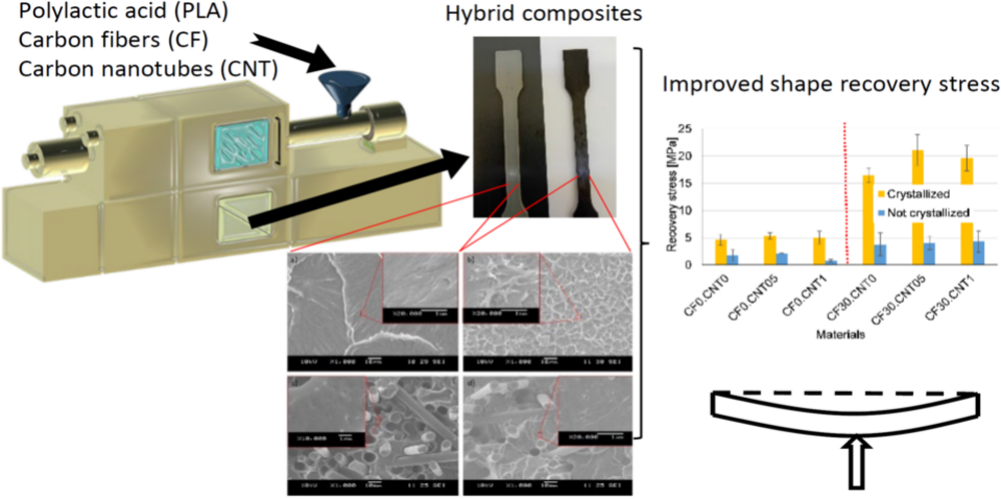

In this study, we showed that hybrid reinforcement—a combination of nanoparticles and fibers—can provide more effective reinforcement for increasing the recovery stress of a shape memory polymer (SMP) than using either filler individually. We mixed carbon fibers (CF) and carbon nanotubes (CNT) into a poly(lactic acid) (PLA) matrix on a twin-screw extruder and injection molded specimen from the hybrid composite. Subsequently, some of the specimens were subjected to crystallizing heat treatment, while others were kept as molded to study the effects of crystallinity as well. We investigated the properties of the specimens with scanning electron microscopy (SEM), differential scanning calorimetry (DSC), and mechanical and thermomechanical tests. We found that the CF helped disperse the CNT properly, allowing them to reinforce more effectively. The CF increased the recovery stress of the samples significantly while decreasing the precision of the recovery due to the rigid nature of the reinforcement. Dispersed CNT could further increase the recovery stress without impairing precision because dispersed CNT formed a deformable reinforcing structure that did not increase elongation at break or plastic strain.

## 1 Introduction

Shape memory polymers (SMPs) are a class of intelligent materials capable of altering their shape in response to an external nonmechanical stimulus. They can be engineered to respond to a wide range of stimuli, the most common being heat.^1^ They are already in mass production for shrink tubes and shrink packaging,^2^ and their role is expected to expand, among other things, into biomedicine, robotics, and control technology.^3,4^

The shape memory effect in polymers is based on a dual structure, where the polymer possesses so-called switches and netpoints. The roles of switches and netpoints can be played by many different structures, like phases or chemical bonds. Switches have to transition from an “open” state that enables molecular movement to a “closed” one that restricts it in response to the stimulus, while netpoints have to remain in place, holding the material together throughout.^5^ One structure that conforms to these requirements is that of semicrystalline polymers. In this case, the amorphous phase plays the role of the switches that respond to the heat stimulus with the glass transition, and the crystalline phase plays the role of the netpoints.^6^

Poly(lactic acid) (PLA) is a semicrystalline polymer that is well suited to shape memory applications because its glass transition temperature (*T*_g_) is around 60 °C, which is practical for many applications; an outstanding material for 3D printing,^7^ it is also biodegradable and based on renewable resources.^8−10^ Thus, a shape memory cycle in the case of PLA begins with heating the material above its *T*_g_, where the amorphous phase transitions to its rubbery phase. Next, the material has to be deformed to the desired programmed shape and cooled below the *T*_g_ while keeping this deformation. Then, the external force can be released; still, some of the internal stress is locked in the material because most of the deformation remains. When subsequently heated above the *T*_g_, the amorphous phase transforms from the glassy state to the rubbery state, and the stored stress is released, returning the SMP to its original shape.^11^ In SMPs, the recovery and shape fixation are never perfect, their precision is characterized using the shape fixity ratio (*R_f_*) and the shape recovery ratio (*R_r_*), as described in eqs 1 and 2.
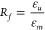
1
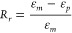
2where *ε*_*m*_ is the maximum strain applied, *ε*_*u*_ is the strain after unloading, and *ε*_*p*_ is the persisting strain after recovery.^12^

SMPs usually exhibit relatively small forces (or stress) during the shape recovery, which means their ability to recover against external forces or move other objects is very limited. This, in turn, limits their applicability, making the increase of recovery stress a research priority.^2,13,14^ Recovery stress (σ_rec_) can be increased by crystallizing heat treatment of injection molded parts.^15^ This can also lead to a decrease in the precision of the recovery. However, crystallizing heat treatment can only go so far, for even greater increases in σ_rec_, fiber reinforcement is commonly used.^16,17^

Nie et al.^18^ investigated the shape memory of PLA samples with three different degrees of crystallinity. They found that higher degrees of crystallinity increased recovery stress by about 50% at most while decreasing the recovery ratio. Nanoparticle reinforcement is also a well-researched method for achieving this and for functionalization of the polymer.^19,20^

Many publications relating to the shape memory of PLA focus on 4D printing, a combination of 3D and shape memory, to manufacture complex devices and geometries.^21,22^ In this case, the focus is often on the precision and activation of the shape memory, not the force. The incorporation of fillers, in this case, is usually to impart some functional property to the material, like electric conductivity or magnetic properties, unlocking new ways of shape memory activation.^23^

In 4D printing, PLA can be reinforced with both bioderived fillers and synthetic ones of nano- and microscale. Natural fillers often swell in water; this phenomenon can offer another switch for shape memory, with the stimulus being water content. The desired functional property for synthetic fillers is most often electric conductivity or magnetism. Reinforcement can also increase the precision of the recovery, acting in a similar way to the crystalline phase.^24,25^

Nowadays, research strives to improve on the basic concepts. Mechanical properties and electrical conductivity can be enhanced even further if continuous fiber reinforcement is applied.^26−28^ Other ways of enhancing the reinforcement include combining PLA with an elastomeric material,^29,30^ treating the reinforcing material for better adhesion,^31^ or plasticizing the PLA to achieve better interaction with the reinforcement.^32^ Liu et al.^29^ reinforced 3D printing PLA filament with 9.26% carbon nanotubes (CNT) and found that the reinforcement increased the recovery force while decreasing the recovery ratio. Still, they achieved recovery ratios above 80%.

A downside to macrofiber reinforcement is that it often makes the recovery slower or less precise. On the other hand, dispersing nanoparticles in a thermoplastic material without degradation can be challenging. Combining macrofibers and nanoparticles in hybrid reinforcement effectively disperses them in the matrix because of the higher shear forces that fibers generate, thus resulting in higher strength.^33^ PLA is highly suitable for the production of such composites.^34^

Liang et al.^35^ investigated the effect of hybrid graphene oxide and carbon fiber reinforcement on epoxy resin. In this case, the nanoparticles were dispersed in solution and later poured onto the fiber cloth. They found that 0.5% graphene reinforcement worked best and increased both the recovery and fixity ratios, as well as the recovery stress, which they explained with improved adhesion with the carbon fibers.

Shape recovery behavior is highly dependent on the vico-elastic properties of the polymer, which is commonly characterized by creep tests. Good creep recovery properties can indicate good shape memory properties.^35,36^ Creep strain can be broken up into instantaneous elastic, viscoelastic, and plastic components that influence shape recovery behavior as well.^37^ However, the literature on the relation of these components and shape memory properties in SMP composites is lacking.

The shape memory properties of hybrid composites have not yet been investigated. Hybrid reinforcement can be more effective than using only one type of reinforcement, and both nano- and microreinforcement can improve the shape memory properties. We theorize that an SMP hybrid composite will have higher σ_rec_ and better shape memory properties than an SMP reinforced with one type of filler exclusively. Therefore, in this research, we combined carbon macrofibers (CF) and carbon nanotubes as reinforcement of an injection molded PLA matrix composite to improve the shape memory characteristics utilizing the synergic effects of the filler combination. We also investigated the effect of the crystallinity on these specimens.

## 2 Materials and Methods

### 2.1 Materials Used

In our experiments, we reinforced Ingeo 2003D (Nature Works Ltd.), a semicrystalline PLA type with 4% d-lactide content. As reinforcement, we combined ZOLTEK PX35 chopped carbon fibers (Zoltek Zrt., Nyergesújfalu, Hungary) and Nanocyl NC 7000 multiwall carbon nanotubes (Nanocyl SA, Sambreville, Belgium). The fibers had a nominal length of 6 mm and a diameter of 7–9 μm; the nanotubes had a length of 0.1–10 μm and a diameter of 10 nm, and their specific surface areas were 250–300 g/m^2^.

### 2.2 Production of Shape Memory Materials

The chopped carbon fibers, the carbon nanotubes, and the matrix were mixed on a Labtech LTE 26-44 twin-screw extruder (Labtech Engineering Co., Ltd., Thailand). The temperature was increased along the screw from 180 to 200 °C in 5 °C increments, and the 26 mm diameter screws had an L/D ratio of 44 and were rotted at a steady speed of 30 1/min. We used a double-hole filament die at 200 °C to make filaments and then pelletized them on a Labtech LZ-120/VS pelletizer (Labtech Engineering Co., Ltd., Thailand). As the hybrid composites were multiscale, a much higher amount of CF than CNT was used, based on the literature. We chose a fiber content of 30% w/w and did not vary it for hybrids, as CF contents of some 10% are usual in the literature.^38,39^ For the CNT content, we chose to investigate two concentrations, 0.5% w/w and 1% w/w, as usually 1–3% contents are used in the literature.^40−42^ With these concentrations plus references, we produced every possible combination.

The pellets were subsequently fed into an Arburg Allrounder 420C injection molding machine (ARBURG GmbH, Germany). We injection molded standard dumbbell specimens of 4 × 10 mm, following the ISO 527 standard, which were later cut to a length of 80 mm to produce a flexural testing specimen (ISO 178). In addition, we injection molded 2 mm thick sheets, which were cut with a saw to produce the specimen for the dynamic mechanical analysis (DMA), as the 4 mm thick samples would have exceeded the DMA’s force limits. For all samples, we set the melt temperature to 210 °C and used the following parameters: injection speed of 44 cm^3^/s, filling pressure of 1500 bar, and packing pressure of 600 bar.

We split each sample into two to investigate the effects of the degree of crystallinity on shape memory and mechanical properties. We applied crystallizing heat treatment on one half at 90 °C for 1 h, leaving the other half as molded. We verified that the heat treatment crystallized the samples using differential scanning calorimetry (DSC). Table 1 lists all the samples prepared.

**Table 1:** Composition and Heat Treatment of Samples

Name	CF [%]	CNT [%]	PLA [%]	Crystallized
CF0.CNT0	0	0	100	no
CF0.CNT05	0	0.5	99.5	no
CF0.CNT1	0	1	99	no
CF30.CNT0	30	0	70	no
CF30.CNT05	30	0.5	69.5	no
CF30.CNT1	30	1	69	no
CF0.CNT0.C	0	0	100	yes
CF0.CNT05.C	0	0.5	99.5	yes
CF0.CNT1.C	0	1	99	yes
CF30.CNT0.C	30	0	70	yes
CF30.CNT05.C	30	0.5	69.5	yes
CF30.CNT1.C	30	1	69	yes

### 2.3 Characterization Methods

#### 2.3.1 Differential Scanning Calorimetry (DSC)

To investigate the crystalline properties, we conducted DSC measurements using a TA Instruments Q2000 DSC (TA Instruments, USA). We cut 5–7 mg of samples and tested them between 20 and 200 °C in a heat–cool–heat cycle. The heating rate was 5 °C/min for both heating and cooling. Based on the literature, the crystalline melting enthalpy of 100% crystalline PLA was taken as 93 J/g.^43^

#### 2.3.3 Flexural Tests

For the assessment of the mechanical properties at room temperature, we conducted flexural tests on the dumbbell specimens. We tested five specimens of each type on a Zwick Z005 universal testing machine (Zwick GmbH., Ulm, Germany) equipped with a 5 kN cell. The test speed was 10 mm/s, and the span between supports was 64 mm. The tests lasted until the specimen broke or reached the conventional deflection, 10% of the span, which was 6.4 mm. We calculated the flexural modulus as the slope of the tangent at the initial near-straight part of the bending curve. We tested 7 specimens of each sample.

#### 2.3.2 Scanning Electron Microscopy (SEM)

We investigated the broken surfaces after the flexural tests on recrystallized samples using a JEOL JSM 6380LA (Jeol Ltd., Japan) scanning electron microscope (SEM). Before inspecting them, we sputtered the samples with a thin gold layer.

#### 2.3.4 Dynamic Mechanical Analysis (DMA)

We conducted dynamic mechanical analysis (DMA) on the samples in a TA Instruments Q800 device (TA Instruments, USA). We cut 2 × 10 × 60 mm samples and put them in a three-point bending clamp with a span of 50 mm. We carried out the test at 1 Hz frequency and 15 μm amplitude while the samples were heated from 30 to 80 °C at a heating rate of 2 °C/min.

#### 2.3.5 Creep Tests

We investigated the strain components in the samples in the same setup as the one later used for shape recovery experiments. We tested them on a Zwick Z0250 universal testing machine (Zwick GmbH., Ulm, Germany) equipped with a 1 kN cell and a heating chamber. During the experiments, the 4 × 10 mm cross-section samples were cut into 70 mm length test specimens and put in a 3-point bending head with a 64 mm span between supports, and they were heated to 70 °C before applying a constant load on the samples. In the case of the crystallized samples, the load was set as 10% of the flexural stress at 2 mm deflection in the flexural tests, while in the case of noncrystallized samples, it was set to 1 N to avoid failure during creep. Noncrystallized samples that did not contain carbon fibers could not be tested without failure. The loading lasted for 15 min, after which the samples were unloaded for 15 min. The deformations were precisely monitored throughout, using a Mercury Monet (Sobriety, Czech Republic) digital image correlation (DIC) device. We tested 3 specimens of each sample.

To investigate the structure of the polymer, we divided the deformation components of the creep deformation according to the Burgers model. The instantaneous elastic strain was defined as the strain that quasi-immediately recovered after unloading. The viscoelastic strain was defined as the strain that recovered after the instantaneous elastic strain before the end of the experiment. The plastic strain was the strain that remained at the end of the experiment.^37^

#### 2.3.6 Free and Constrained Recovery Experiments

We evaluated the shape memory capabilities of the samples in both free recovery and constrained recovery cycles, conducted with the same Zwick Z0250 universal testing machine (Zwick GmbH., Ulm, Germany) equipped with a 1 kN cell and a heating chamber. During the experiments, the 4 × 10 mm cross-section samples were cut to 70 mm length test specimens and put in a 3-point bending head with a 64 mm span between supports. The samples were heated in the heating chamber at 70 °C for 5 min, where they were deformed to a 2 mm deflection (ε_m_). Keeping the crosshead in place, they were taken out of the heating chamber and cooled at room temperature for 5 min. Subsequently, they were put back into the heating chamber and kept there for 5 min while the recovery took place. We tested 3 specimens of each sample.

In the case of the free recovery cycles, the deformations were precisely monitored using a Mercury Monet (Sobriety, Czech Republic) type digital image correlation (DIC) device. The samples were painted to be better visible for the DIC, and the deflection was monitored using a single-point probe in the middle of the specimen. From the deflection data, we calculated the shape fixity and recovery ratios using eqs 1 and 2.

In the case of constrained recovery cycles, we kept a constant deflection of 0.01 mm in place during recovery. and monitored the force on the crosshead with the cell. We then calculated σ_rec_ from the maximum force.

## 3 Results and Discussion

### 3.1 Scanning Electron microscopy (SEM)

**Figure 1:**
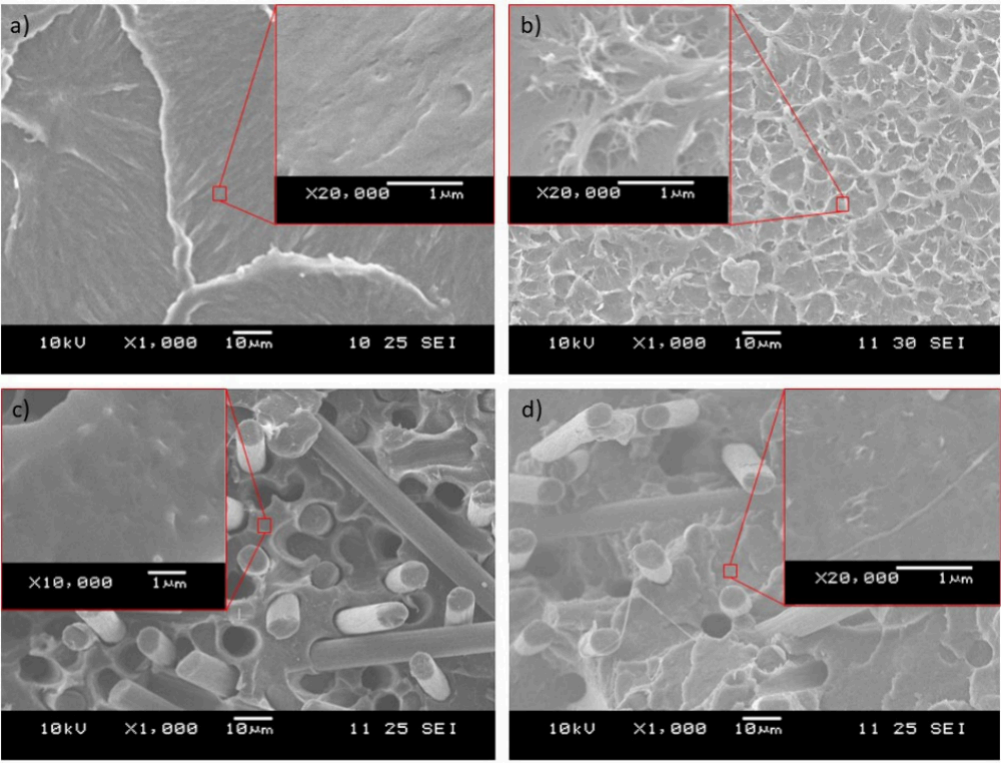
Characteristic SEM images of the break surfaces for the (a) CF0.CNT0 sample, (b) CF0.CNT1 sample, (c) CF30.CNT0 sample, and (d) CF30.CNT1 sample.

The SEM images show that the addition of the CF helped distribute the CNT and break up agglomerates (Figure 1). The addition of the CF leads to higher shear forces being generated during extrusion, with high forces that can help break up nanoparticle agglomerates.^44^ CF0.CNT1 (Figure 1b) showed a morphology like the one in the study of Liu et al.,^23^ with the CNT particles sticking to each other. In our case, not all of the cross-section was covered with CNT, probably because of the difference in the amount used, 1% in our case and 9% in theirs. On other parts of the surface, very few nanotubes could be observed. In the case of the CF30.CNT1 sample (Figure 1d), no agglomerates could be seen, but instead, CNT was distributed between the fibers.

Comparing Figure 1c and Figure 1d, we can see that the hybrid composites produced a different fracture surface compared to carbon fiber composites. In the case of hybrid composites, the visible fiber lengths are shorter, which is a sign of good adhesion, as the fibers broke rather than pulled out. There are also differences in the surrounding matrix. In the case of hybrid composites, the broken surface is more structured, which indicates that the CNT reinforced the matrix, made the stress distribution more homogeneous, and transferred the stress to the fibers more effectively.

In the case of the CF0.CNT0 (Figure 1a) and CF30.CNT0 (Figure 1c) samples no structure like what we see in the CNT-containing samples can be observed. Thus, we can conclude that the mixing was successful and that the structureswe see in Figure 1b and d are indeed nanotubes.

### 3.2 Differential Scanning Calorimetry (DSC)

**Figure 2:**
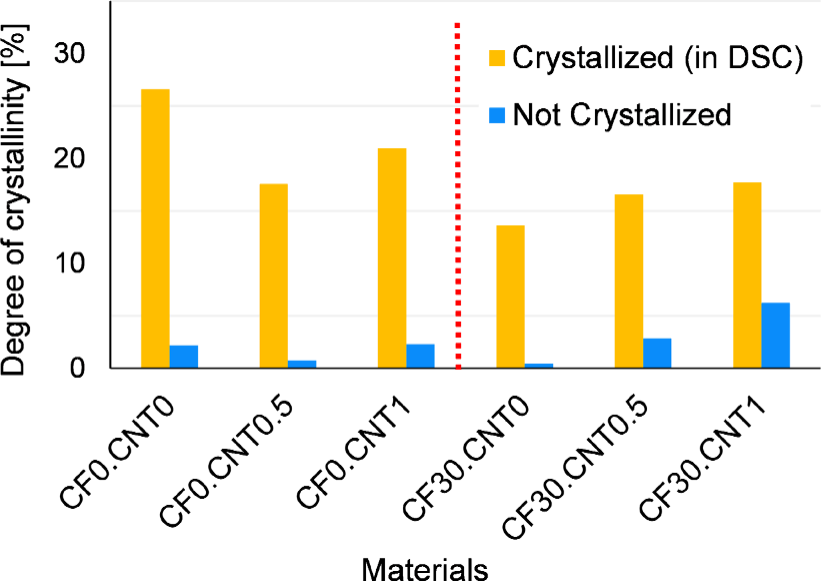
Degrees of crystallinity from DSC before and after crystallizing heat treatment.

The effectiveness of the crystallizing heat treatment is clearly visible from the DSC results (Figure 2). All crystallized samples had at least twice the degree of crystallinity than their noncrystallized counterparts, which never had more than 10% crystallinity.

The CNT was decisively able to act as a nucleating agent when combined with the CF; when present alone, its effects were less clear. When the samples had no CF, the degree of crystallinity was somewhat reduced compared to the reference, likely related to the CNT aggregates. The addition of CF reduced the degree of crystallinity, as it restricted molecular movement. The hybrid composites had higher degrees of crystallinity than samples with CF alone, likely as a result of the nucleating properties of distributed CNT.

### 3.3 Flexural Tests

**Figure 3:**
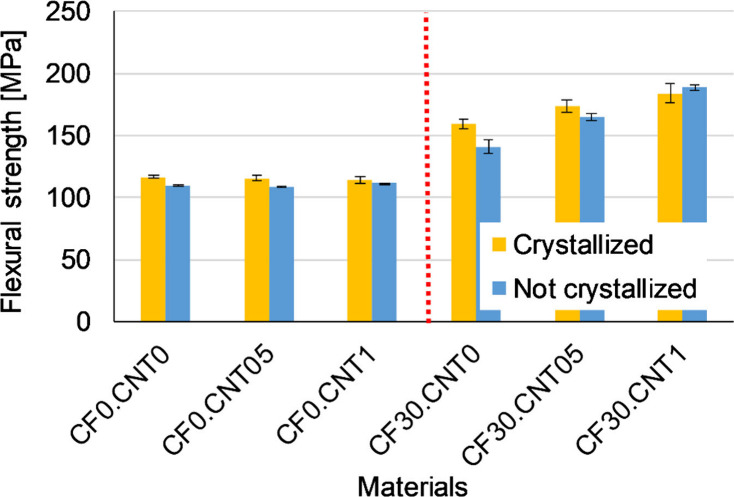
Average flexural strength of samples.

The flexural tests showed the effects of the morphology on the mechanical properties. The CF substantially increased the strength of the material (Figure 3). Crystallizing heat treatment increased strength to a much lesser degree, with standard deviations sometimes overlapping. CNT, when not combined with CF, had no substantial effect on strength. However, when properly dispersed by the increased shear from the presence of the fibers, the CNT could reinforce effectively, increasing the strength significantly.

**Figure 4:**
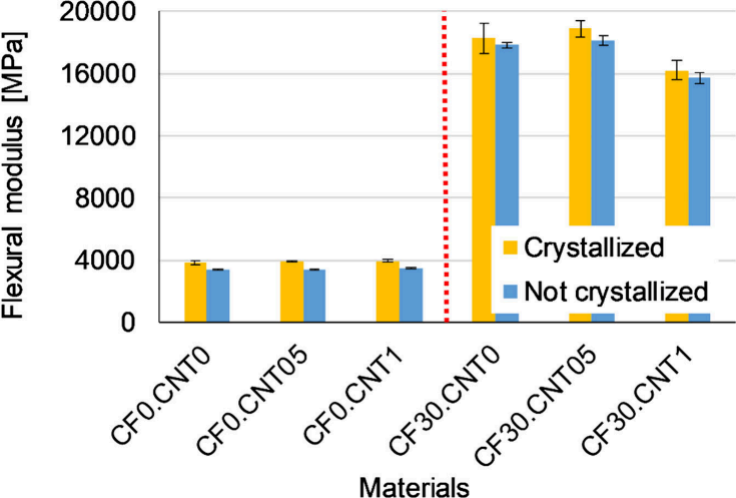
Average flexural modulus of samples.

The effect of CF on the flexural modulus (Figure 4) was more pronounced than on strength, increasing the modulus by several folds. The presence of CNT could not further increase the modulus, which means that for small deformations, the influence of the carbon fibers was dominant. For the hybrid composite containing 1 wt % CNT, the modulus decreased slightly compared to the CF30.CNT05 sample, but it is still outstanding from an engineering point of view. This could be due to the fact that all nanotube aggregates could remain in the system, which did not participate in the load bearing.

**Figure 5:**
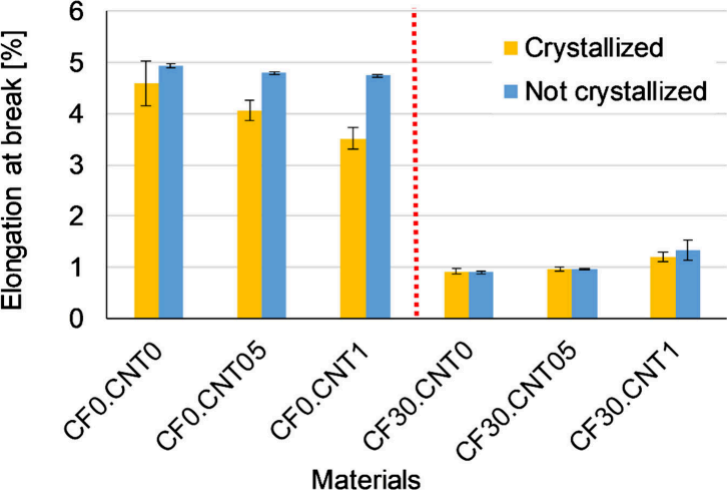
Average elongation at break of samples from flexural tests.

CF reinforcement greatly decreased the elongation at break (Figure 5), while CNT had much less of an effect on the material’s ability to deform. The CNT had a more complex effect on elongation at break, as it was dependent both on the crystallizing heat treatment and on the distribution of the CNT particles by hybrid reinforcement. CNT reinforcement alone decreased elongation at break slightly, as CNT aggregates could act as weak points to start fractures, this effect was exacerbated by the heat treatment, which made the material more rigid. The better distributed CNT particles in the case of the CF30.CNT1 sample increased elongation at break somewhat.

### 3.4 Dynamic Mechanical Analysis (DMA)

**Figure 6:**
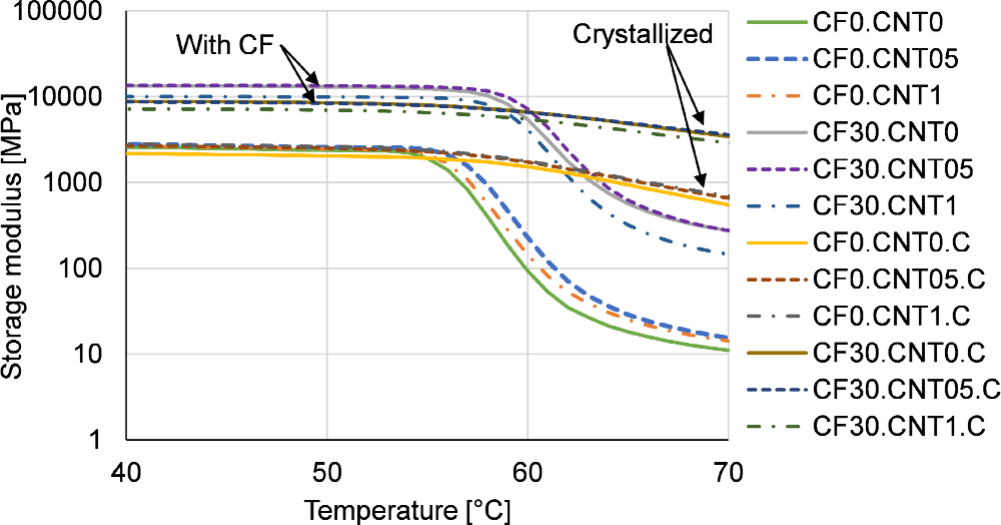
Glass transition region as seen on DMA curves.

The results of the DMA measurements align with the modulus results of the bending tests (Figure 6). The samples containing CF had a much larger storage modulus, while the effects of crystallization and CNT were negligible. An additional effect of the CF reinforcement is that the *T*_g_ increased by about 3 °C, from around 59 °C to around 62 °C. This shows that the fibers reduced molecular mobility, leading to decreased elongation at break. The most prominent effect of crystallization is that above the *T*_g_, the storage modulus remained much higher; in this respect, crystallinity had a larger effect than even the CF.

### 3.5 Creep Tests

**Figure 7:**
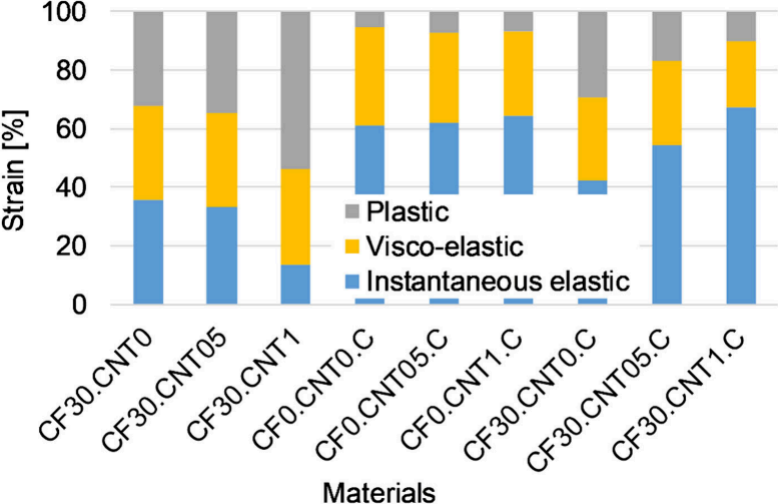
Deformation components for different samples.

During the creep tests, the total deflection of most of the samples was between 1 and 4 mm, so it was not significantly different from the deflection programmed during shape memory (2 mm). The results of the creep tests showed that the crystallizing heat treatment increased the fraction of the instantaneous elastic strain while decreasing that of the residual strain (Figure 7). The fraction of the viscoelastic strain was about the same for all samples, around 30%. The addition of CF significantly increased the fraction of residual strain while decreasing that of the instantaneous elastic strain. The rigid fibers can break or pull out under strain, which is unrecoverable. Undistributed CNT had a small effect on the components of strain. It slightly increased the fraction of residual strain and decreased that of the instantaneous elastic and viscoelastic components. This is because the CNT aggregates can not recover strain like the polymer molecules surrounding them.

**Figure 8:**
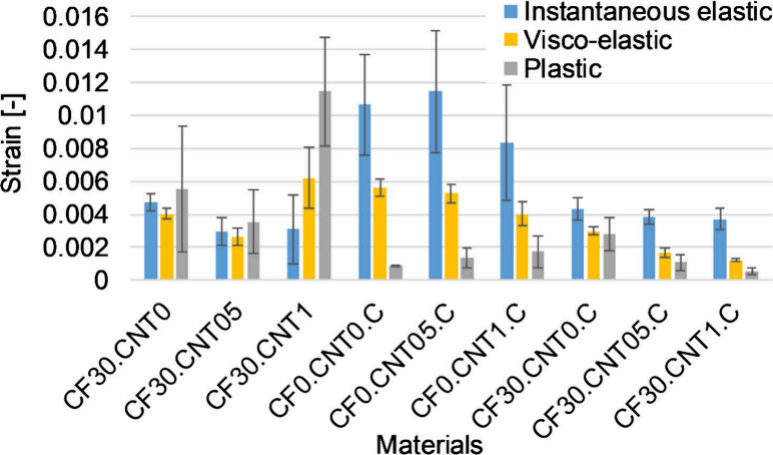
Deformation components as absolute values for different samples.

**Figure 9:**
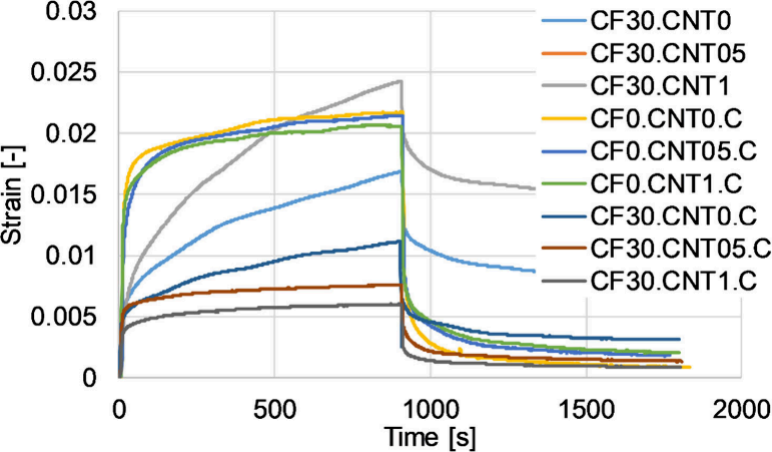
Representative creep curves for different samples.

When distributed by the carbon fibers, the CNT had a greater effect. In the case of the noncrystallized samples, particularly CF30.CNT1, the plastic strain increased significantly at the expense of the instantaneous elastic strain; the overall strain also increased significantly, so the creep resistance decreased (Figure 8, Figure 9). This may be the effect of the residual aggregates present at high nanotube content, which is consistent with the bending modulus and storage modulus. In the case of crystallized samples, the dispersed CNT decreased the viscoelastic and residual strains while keeping the instantaneous elastic strain about the same. In this case, the crystallization-increasing effect of the CNT overwrote the effect of the aggregates.

### 3.6 Free Recovery Experiments

**Figure 10:**
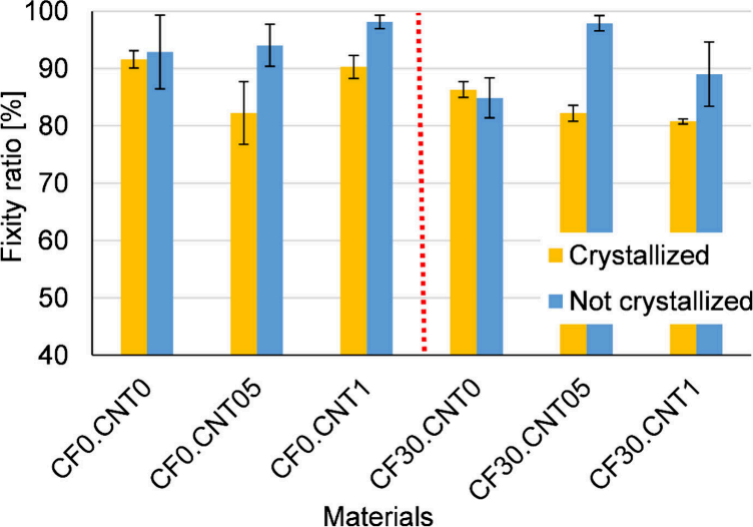
Fixity ratios from free recovery experiments.

In the free recovery experiments, all samples showed good shape memory properties, with almost all samples displaying a fixity and recovery ratio of above 80% (Figure 10). In most cases, the results of the noncrystallized samples showed a higher standard deviation than their crystallized counterparts, as the properties in this case were much more sensitive to inhomogeneity in the samples.

In many cases, the shape fixity ratios for noncrystallized samples were higher than those of crystallized samples. This is because, during unloading, the glassy amorphous phase keeps the polymer from recovering its shape. The creep tests also showed higher residual strain, which causes the same effect. As this phase took up more of the volume in these samples, they were more successful at this.

**Figure 11:**
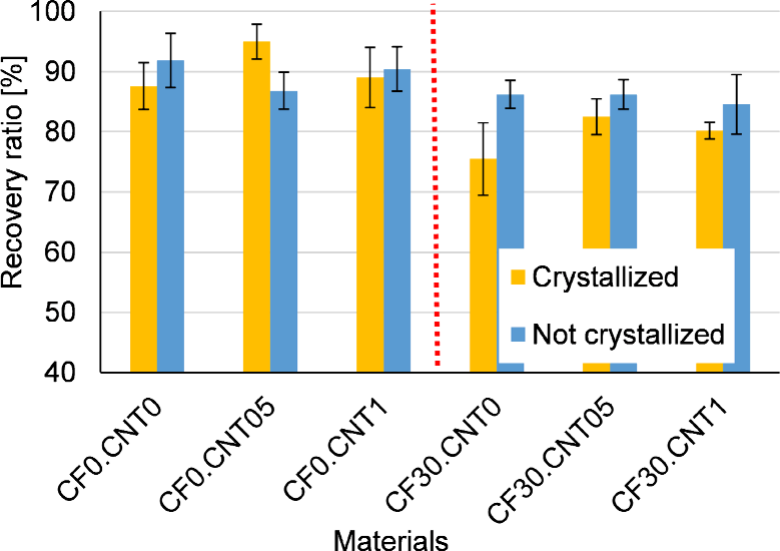
Recovery ratios from free recovery experiments.

However, the increased fraction of the crystalline phase did not improve the precision of the recovery (Figure 11). Generally, the standard deviations for crystallized and noncrystallized recovery ratios greatly overlap despite the fact that in creep experiments, noncrystallized samples had significantly more plastic strain. This is because of the increased time-dependent properties of the noncrystallized samples; in the longer creep test, the plastic strain could build up over time, while in the recovery tests, they were quickly cooled down. The presence of the CF made both shape fixity and shape recovery less precise on average. As shown previously, the fibers impeded molecular movement and thus caused higher amounts of residual strain. The presence of CNT could not be said to conclusively influence the precision of the shape memory cycle in either direction, although when distributed and combined with crystallizing heat treatment, it slightly improved shape recovery and decreased shape fixity, in line with the observations on the residual strain.

### 3.7 Constrained Recovery Experiments

**Figure 12:**
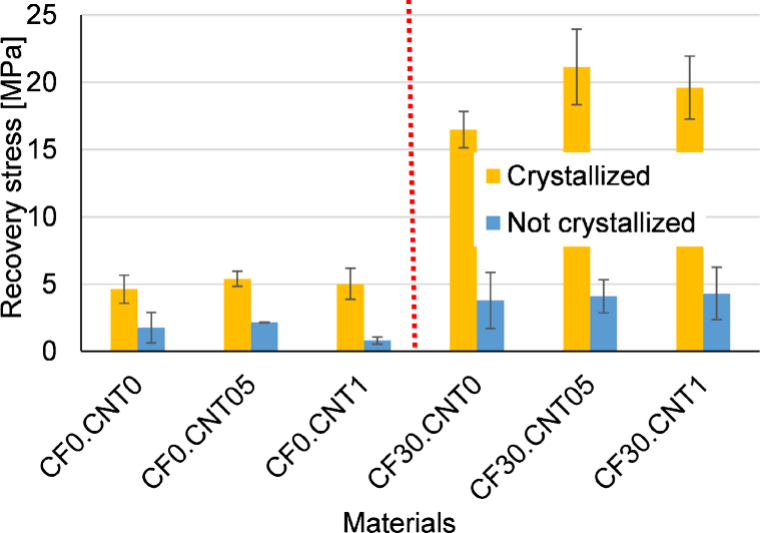
Recovery stress from constrained recovery experiments.

In the constrained recovery experiments, both the crystallizing heat treatment and the addition of the CF clearly showed its effect (Figure 12). Noncrystallized samples had low recovery stresses, around 1–2 MPa, but the crystallization raised this to around 5 MPa. This increase is much larger than any change in mechanical properties measured at room temperature, indicating that room temperature measurements do not predict the recovery stress very well; the several-fold increases seen on the DMA curves above T_g_ must be considered as well. The addition of the CF increased the recovery stress to about 4 times in the case of crystallized samples and to about twice the value in the case of the noncrystallized samples. The increase is in line with the increase in the modulus both above and below the T_*g*_ for crystallized samples, but it is quite low for noncrystallized samples.

Like in previous experiments, the CNT failed to reinforce effectively when left in agglomerates. CNT agglomerates had no significant effect in crystallized samples, but in the case of the noncrystallized CF0.CNT1 the CNT decidedly decreased the recovery stress. This can be explained by the improved heat conductivity of the CNT; because of this, the amorphous phase could have become more rubbery and unable to store stress.

However, properly dispersed, the CNT increased the recovery strength effectively. In the case of crystallized samples, we see clear and large increases in recovery stress from 17 to 22 MPa, which are more substantial than CNT’s effect on room temperature mechanical properties. The recovery stress decreased slightly for 1 w/w% CNT reinforcement because of the agglomeration of the nanotubes.

To get an even clearer picture of the constrained recovery, we evaluated the stress–strain curves from shape programming as well, effectively treating them as a high-temperature flexural test (Table 2). With this kind of analysis, we can see that the noncrystallized samples had much higher standard deviations, especially in comparison to the average values, again because of their greater susceptibility to inhomogeneity in the samples. The reason why the noncrystallized samples had lower recovery stress is not that they had lower programming stress or modulus but because they were much less able to store this stress internally and release it later, which is a function in large part attributed to the crystalline phase. This ability to store the stress is characterized here by the programming stress ratio, which is the programming stress divided by the recovery stress. This ratio explains entirely the about 4 times difference between crystallized and noncrystallized samples. This also corresponds with the decreases in residual strain in creep tests.

**Table 2:** Relation between Recovery Stress and Programming Stress

Name	Recovery stress [MPa]	Programming stress [MPa]	Programming modulus [MPa]	Programming stress ratio [-]
CF0.CNT0	1.76 ± 1.1	10.97 ± 8.2	1298 ± 715	0.16
CF0.CNT05	2.15 ± 0.0	9.92 ± 6.9	1142 ± 806	0.22
CF0.CNT1	0.81 ± 0.3	5.09 ± 3.8	886 ± 309	0.16
CF30.CNT0	3.79 ± 2.1	35.84 ± 18.8	3328 ± 741	0.11
CF30.CNT05	4.10 ± 1.2	35.74 ± 8.8	7674 ± 1249	0.11
CF30.CNT1	4.31 ± 1.9	31.75 ± 20.8	6505 ± 3045	0.14
CF0.CNT0.C	4.62 ± 0.8	6.47 ± 1.1	543 ± 188	0.71
CF0.CNT05.C	5.39 ± 0.6	7.02 ± 0.6	557 ± 60	0.77
CF0.CNT1.C	5.02 ± 1.7	7.20 ± 1.2	613 ± 135	0.70
CF30.CNT0.C	16.47 ± 2.1	36.37 ± 1.4	4922 ± 318	0.45
CF30.CNT05.C	21.12 ± 2.0	47.16 ± 2.8	5584 ± 334	0.45
CF30.CNT1.C	19.60 ± 2.7	42.68 ± 2.3	4907 ± 590	0.46

The programming stress ratio also highlights why CNT reinforcement, in this case, was more efficient than in the flexural tests. The addition of CF, while increasing the programming stress and modulus, also decreased the ratios at which these were stored. On the other hand, the dispersed CNT reinforcement managed to increase the programming stress and modulus while leaving the ratio at which it is stored unaltered, achieving a more effective reinforcement. This is because the CF reinforcement created more residual strain, thus resulting in less ability to store stress, while dispersed CNT decreased the residual strain, allowing more stress to be recovered.

## 4 Conclusions

In this study, we investigated the effects of hybrid reinforcement on the shape memory of injection-molded PLA. The CF helped distribute the CNT agglomerates, which had a visible effect on break surfaces investigated with SEM, and the distribution led to good reinforcement and higher strength. The crystallizing heat treatment greatly increased the degree of crystallinity, which was also increased by the dispersed CNT. The samples showed good precision during shape recovery. Both the fixity and recovery ratios were above 80% in almost all cases. Crystallinity decreased the fixity ratio while increasing the recovery ratio, owing to the role of the crystalline domains as netpoints during shape memory and higher residual stress for noncrystalline samples. CF reinforcement decreased both ratios because of the rigidity of the fibers, while CNT did not have a clear effect in either direction.

The recovery strength increased considerably when the samples were crystallized or reinforced with CF. Dispersed CNT had a somewhat smaller effect on the recovery stress, and nondispersed CNT had almost none. In the case of crystallinity, this increase was due to a better ability to store and recover the programming stress. For CF, the increases in programming stress led to higher recovery stress, while the ability to store and recover said stress was undermined by high residual strains. Dispersed CNT was able to raise the programming stress while decreasing the residual strain, thus not impairing the ability to store and recover the internal stress. The addition of CNT into the composite improved the recovery stress significantly while not affecting any properties adversely, while the same cannot be said for CF. Achieving the desired recovery stress with hybrid reinforcement is thus more effective than relying only on CF.
